# GOBO: Gene Expression-Based Outcome for Breast Cancer Online

**DOI:** 10.1371/journal.pone.0017911

**Published:** 2011-03-21

**Authors:** Markus Ringnér, Erik Fredlund, Jari Häkkinen, Åke Borg, Johan Staaf

**Affiliations:** 1 Department of Oncology, Clinical Sciences, Lund University and Skåne University Hospital, Lund, Sweden; 2 CREATE Health Strategic Center for Translational Cancer Research, Lund University, Lund, Sweden; Baylor College of Medicine, United States of America

## Abstract

Microarray-based gene expression analysis holds promise of improving prognostication and treatment decisions for breast cancer patients. However, the heterogeneity of breast cancer emphasizes the need for validation of prognostic gene signatures in larger sample sets stratified into relevant subgroups. Here, we describe a multifunctional user-friendly online tool, GOBO (http://co.bmc.lu.se/gobo), allowing a range of different analyses to be performed in an 1881-sample breast tumor data set, and a 51-sample breast cancer cell line set, both generated on Affymetrix U133A microarrays. GOBO supports a wide range of applications including: 1) rapid assessment of gene expression levels in subgroups of breast tumors and cell lines, 2) identification of co-expressed genes for creation of potential metagenes, 3) association with outcome for gene expression levels of single genes, sets of genes, or gene signatures in multiple subgroups of the 1881-sample breast cancer data set. The design and implementation of GOBO facilitate easy incorporation of additional query functions and applications, as well as additional data sets irrespective of tumor type and array platform.

## Introduction

Breast cancer is one of the most common malignancies in the world. It is evident that breast cancer is a heterogeneous disease characterized by different tumor biological and histological subtypes, age of onset, clinical course and responsiveness to treatment. Although currently used prognostic and treatment-predictive markers for breast cancer have markedly improved treatment decisions, additional markers are required to further tailor treatment for individual patients.

Microarray-based gene expression profiling has been used extensively to characterize the transcriptome of breast cancer, resulting in the identification of new molecular subtypes and markers or signatures of potential therapeutic and prognostic importance [1]–[12]. Although microarray-based gene expression profiling of breast carcinoma holds promise for future improvement in treatment decisions, current studies aimed at deriving prognostic or treatment predictive gene signatures are often limited by a low number of included patients, typically tens to hundreds, due to cost and availability of suitable tissue specimens. The growing accumulation of data from prognostic and/or treatment predictive gene expression studies on breast cancer in public repositories, e.g. Gene Expression Omnibus [13], provides an opportunity to construct pooled gene expression data sets comprising a larger number of patients with sufficient clinical data. Pooled gene expression data sets of different sizes have been used for prognostic validation of single and multigene predictors [14]–[17], identification of gene set modules associated with prognosis [7], [18]–[20], distinction of clinical types of breast cancer [21], and dissection of underlying processes in reported prognostic predictors [19], [22]. Although data sets in larger pooled sets are often individually available through public repositories, the combined pooled data sets are commonly not available, thereby representing an obstacle to researchers with limited bioinformatic resources. To address this latter shortcoming, we developed Gene expression-based Outcome for Breast cancer Online (GOBO). GOBO is aimed at providing an online tool for prognostic validation of single genes, sets of genes or simple predictors in a pooled breast cancer data set comprising 1881 cases from eleven public data sets analyzed using Affymetrix U133A arrays. In addition, GOBO allows online investigation of the expression of single genes or sets of genes across a large set of commonly used breast cancer cell lines [23], as well as identification of co-expressed genes in both breast cancer cell lines and breast tumors. Using the *CCNB1* gene and a Core Serum Response gene signature as two examples we illustrate the usefulness of GOBO for rapid online analysis in a large breast cancer data set

## Results

### Overview of GOBO applications

**Figure 1:**
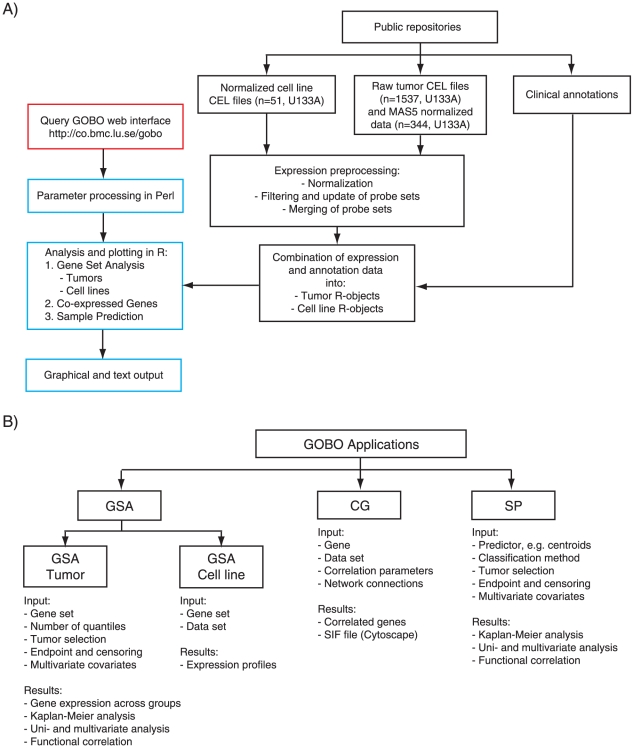
Architecture of GOBO. (A) Flowchart of the GOBO software illustrating the three different modules, data set module (black), web interface module (red) and analysis module (blue). (B) Layout of GOBO applications with respect to input and results generated.

The web interface of GOBO allows precompiled data sets to be queried by the three main applications of GOBO: Gene Set Analysis (GSA), Co-expressed Genes (CG), and Sample Prediction (SP) (Figure 1). Currently, the precompiled data sets consist of gene expression data and annotation data for a pooled 1881-sample breast tumor set and 51 previously reported breast cancer cell lines [23]. The 1881-sample breast tumor set comprises 11 public data sets (Table 1) analyzed using Affymetrix U133A arrays and processed as described (in [15] and File S1). GSA is further divided into outcome analysis in breast tumors (GSA-Tumor) and expression patterns in breast cancer cell lines (GSA-Cell line). In both GSA applications the input is either a single gene or probe identifier, or a set of gene/probe identifiers (referred to as a gene set hereinafter). CG allows identification of co-expressed genes by provision of a single gene identifier in both the breast tumor data set and the panel of breast cancer cell lines. SP allows users to investigate the association of their classifiers (in certain predefined forms) with outcome in the 1881-sample breast cancer set.

**Table 1:** Clinical characteristics of individual data sets in the combined 1881-sample Affymetrix U133A data set.

GEO ID	Number of samples	ER:−/+^A^	LN:−/+^B^	DMFS (0/1)^C^	Average DMFS (years)	OS(0/1)^D^	Average OS (years)	RFS^E^ (0/1)	Average RFS (years)	Grade:1/2/3	Median age (years)	Average size (mm)	Reference
GSE7390	198	64/134	198/0	136/62	10.8±5.4	142/56	11.4±3.7	107/91	9.3±5.6	30/83/83	46±7	22±8	[50]
GSE3494	251	34/213	158/84	NA	NA	132/119	7.9±4.1	155/96	5.5±3.4	67/128/54	64±14	22±13	[51]
GSE1456	159	29/130	94/60	NA	NA	119/40	6.4±1.9	119/40	6.2±2.3	28/58/61	56±14	22±12	[52]
GSE2034	286	77/209	286/0	179/107	6.5±3.5	NA	NA	NA	NA	6/42/139*	53±12*	10±6*	[6]
GSE2603	99	42/57	34/65	55/27	5.2±2.3	NA	NA	NA	NA	NA	56±14	36±17	[53]
GSE6532	327	45/262	221/85	225/68	6.3±3.7	NA	NA	195/111	6.3±3.7	65/145/60	60.5±12	23±12	[27]
GSE4922	40	NA	NA	NA	NA	NA	NA	NA	NA	0/40/0	NA	NA	[36]
GSE12093	136	0/136	136/0	116/20	7.7±3.2	NA	NA	NA	NA	NA	NA	NA	[28]
GSE5327	58	58/0	NA	47/11	6.8±3.1	NA	NA	NA	NA	NA	NA	NA	[54]
GSE11121	197	NA	197/0	153/44	7.8±4.2	NA	NA	NA	NA	29/135/33	NA	21±10	[55]
Chin	130	46/84	59/71	102/27	5.7±4	84/45	6.4±3.7	NA	NA	14/46/65	51±15	27±14	[41]
Total	1881	395/1225	1383/365	1013/366	7.2±4.2	477/260	8.2±4.4	576/338	6.7±4.2	239/677/495	55±13	20±12	

A ER: Estrogen receptor.

B LN: Lymph node.

C DMFS: Distant metastasis-free survival.

D OS: Overall survival.

E RFS: Relapse-free survival.

*Collected from publications.

Pooling of gene expression data sets generated at different institutions and time points may introduce artifacts due to, e.g., different reagent lots, scanner settings, RNA extraction procedures, sample selections and laboratory practices. To investigate whether such data set related artifacts existed in the 1881-sample set we undertook three investigations. First, we performed principal component analysis (PCA) and analyzed associations between principal components and a variety of sample annotations (Figure S1 in File S2). We observed that the different data sets are contributing variation, however, in combination with biological/clinical features. Importantly, the first three components are significantly associated with molecular subtypes, ER status, histological grade and lymph node status. Since some of the included data sets contain only ER-negative, ER-positive or node negative tumors, respectively, variation associated with data sets cannot be corrected for without the risk of deleteriously affecting biological/clinical features. Second, we examined gene expression levels of Affymetrix probes suggested from literature to provide high sensitivity and specificity in assigning cases to clinical subtypes based on ER and HER2-status. Expression levels were investigated in nine of eleven data sets for samples stratified by ER-status and PAM50 gene expression subtype classes (Figures S2 to S4 in File S2). These analyses show consistency across data sets in the expression of ER and HER2 across samples clinically annotated with ER-status, as well as in what would be expected for the PAM50 gene expression subtypes. Finally, we performed hierarchical clustering of the 1881-sample data set using the PAM50 gene set [24], showing that the different data sets are intermixed (Figure S5 in File S2). Taken together, these validations indicate that the 1881-sample set can be used for the types of supervised analyses made available through GOBO. Moreover in the GOBO applications, we provide analysis that reveals the influence of each data set on the result.

### Association of gene sets with breast cancer outcome (GSA-Tumor)

Numerous studies have investigated the prognostic importance of mRNA expression, as measured by microarrays or quantitative PCR, from single genes or sets of genes in different breast cancer subgroups [14], [18], [19], [25]. An online tool was recently reported that allows Kaplan-Meier survival analysis of single genes in a pooled 1809-sample microarray-based breast cancer data set [26]. The GSA-Tumor application of GOBO extends on the application reported by Gyorffy et al. [26] by allowing: 1) expression of gene sets to define patient cohorts based on a larger number of quantiles, 2) gene weights to be used in computation of a combined expression score, 3) time-censoring, 4) multivariate analysis of gene expression groups with other covariates such as estrogen receptor (ER) status, lymph node (LN) status, histological grade, stratified tumor size (≤20 mm or >20 mm), stratified patient age (≤50 years or >50 years), 5) analysis of gene set expression across clinical (ER, LN, and histological grade) and molecular breast cancer subgroups [1], [24], and 6) correlation of expression of individual genes in a gene set with co-expressed gene modules emulating breast cancer specific biological processes (Fredlund et al., Manuscript in preparation).

In GSA-Tumor a gene set comprises either a single or multiple genes. Weights can be associated with each gene, thus allowing genes with positive and negative relative expression levels to be combined into a gene set. In the case of multiple genes an averaged gene set expression (including weights if supplied) is computed, prior to dividing the data set into patient cohorts based on gene expression quantiles. Association with outcome is investigated for stratified patient cohorts using distant metastasis-free survival (DMFS), relapse-free survival (RFS) or overall survival (OS) in different breast cancer subgroups. GSA-Tumor allows division of the 1881-sample data set into five major subgroups based on clinical annotations available from GEO [13]; 1) all 1881 tumors, 2) ER-positive tumors (n = 1225), 3) ER-negative tumors (n = 395), 4) systemically untreated patients (n = 927), and 5) patients treated with tamoxifen alone (n = 326, GSE6532 [27] and GSE12093 [28]). In each of the selected major subgroups, survival analysis including Kaplan-Meier, univariate and multivariate analysis, is performed on subsequently more stratified groups based on gene expression subtypes (basal-like, HER2-enriched, luminal A, luminal B, normal-like) [24], [29], ER-status, LN-status, histological grade, and treatment status (untreated or tamoxifen treated tumors).

**Figure 2:**
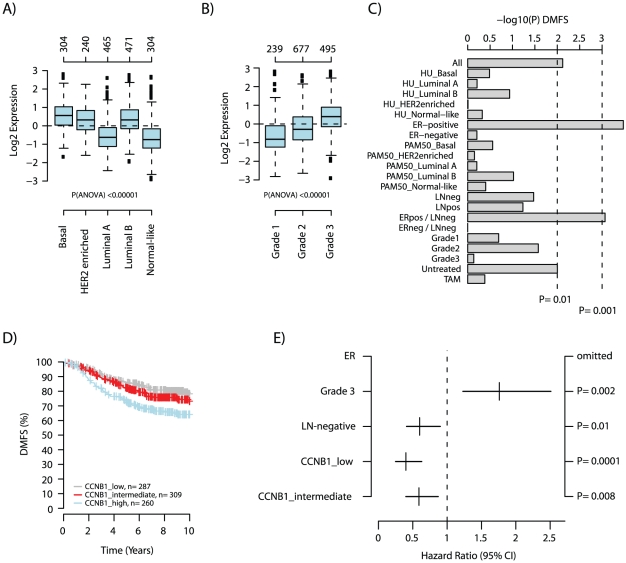
GSA-Tumor analysis of *CCNB1* using the 1881-sample breast cancer data set. (A) Box plot of *CCNB1* gene expression for tumor samples stratified according to PAM50 subtypes [24]. (B) Box plot of *CCNB1* gene expression for tumor samples stratified according to histological grade. (C) Association with outcome for *CCNB1* gene expression levels in subgroups of breast cancer using DMFS as endpoint and 10-year censoring. Samples in the 1881-sample set were stratified into three quantiles based on *CCNB1* expression, *CCNB1*_low (log2 expression −2.9 to −0.497), *CCNB1*_medium (−0.48 to 0.416), and *CCNB1*_high (0.42 to 2.8) followed by Kaplan-Meier survival analysis in 21 subgroups for 1379 cases with DMFS follow-up. Logrank P-values are shown as −log10(P-value). (D) Kaplan-Meier analysis, using DMFS as endpoint, for ER-positive tumors (n = 856) stratified into the three quantiles based on *CCNB1* gene expression level. (E) Corresponding multivariate analysis for ER-positive tumors (n = 554) using lymph node status and stratified histological grade (histological grade 1 and 2 vs. 3) as covariates and DMFS as endpoint with 10-year censoring.

Proliferation has been recognized as one of the key prognostic factors in breast cancer, and has been found to be the major constituent of several prognostic gene expression signatures [19], [22]. Aberrant cell proliferation requires deregulation of several cellular functions or pathways, including cell cycle progression. Cyclins and cyclin-dependent kinases (CDKs) are key regulators of the cell cycle, controlling the progression and initiation of different phases of the cell cycle [30]. One of the key components of the transition between the G2 and M phase in the cell cycle is the protein complex formed by cyclin B1 (*CCNB1*) and CDK1 [31]. CCNB1 protein levels have been shown to differ between breast cancer subgroups [32], tumors of different histological grade [32], [33], and to be associated with breast cancer outcome [32]–[34]. In addition, *CCNB1* is also included in several prognostic gene signatures such as the 21-gene recurrence score [10] and two genomic grade signatures [35], [36]. Using GSA-Tumor we stratified the 1881-tumor set into three quantiles based on *CCNB1* gene expression (File S1). GSA-Tumor analysis of *CCNB1* gene expression shows, in agreement with previous protein-based studies, that elevated expression is associated with the basal-like and luminal B gene expression subgroups (Figure 2A), higher histological grade (Figure 2B), and worse clinical outcome predominantly in ER-positive breast cancers (Figures 2C and D) supported by multivariate analysis (Figure 2E) [32]–[34].

### Gene set expression in breast cancer cell lines (GSA-Cell line)

Cancer cell lines, derived from immortalized cancer cells, are commonly employed in cancer research as model systems for functional assessment of aberrant gene function and prediction or indication of response to therapeutic substances. Furthermore, it has been shown that breast cancer cell lines in many aspects mirror the heterogeneity in transcriptional and genomic aberrations found in breast tumors [23], [37], underlining the importance of selecting the appropriate cell line for a specific experiment. The GSA-Cell line application provides an interface for exploring mRNA expression levels of gene sets across 51 previously reported breast cancer cell lines [23], reporting both relative gene expression levels across the cell line panel, as well as summarized average intensity values. Similarly as for GSA-Tumor, if a gene set consists of multiple genes an average expression is computed for the total gene set, taking consideration to gene weights if supplied.

**Figure 3:**
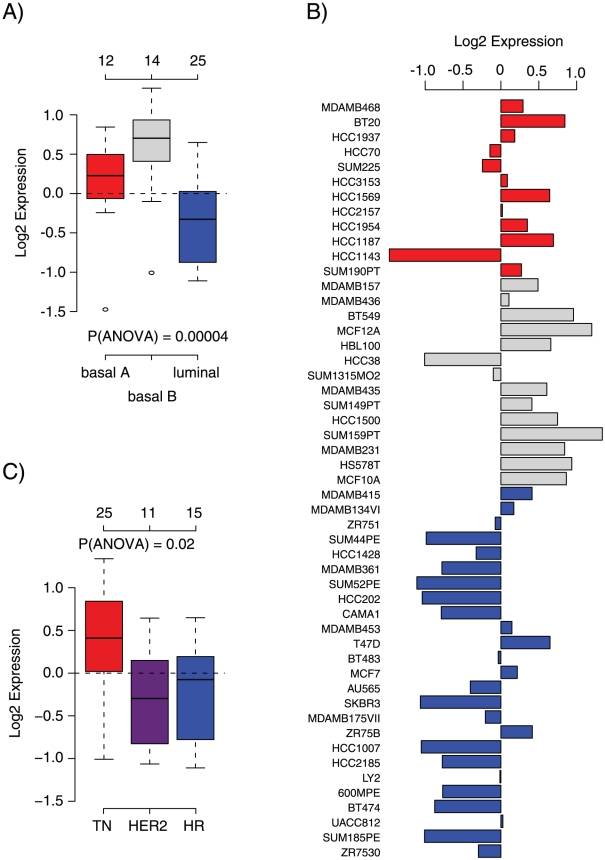
Result of GSA-Cell line analysis for *CCNB1*. (A) Box plots of *CCNB1* gene expression across cell lines grouped in the basal A (red), basal B (grey) and luminal (blue) subgroups [23]. (B) Expression of *CCNB1* across the 51 individual cell lines. Colours according to (a). (C) Box plot of gene expression for *CCNB1* across cell lines grouped into clinical subtypes; triple negative (TN, red), HER2-positive (HER2, purple), and Hormone receptor-positive (HR, blue) based on annotation data from Neve et al. [23].

Using the GSA-Cell line application, we investigated *CCNB1* mRNA expression levels across the breast cancer cell line panel. Highest gene expression was observed in the basal B subgroup associated with a more stem-cell like phenotype and recently also the claudin-low subtype [23], [38], followed by the basal A and luminal subgroups as defined by Neve et al. [23] (Figures 3A and B). In line with previous reports analyzing CCNB1 protein expression we found that triple-negative cell lines displayed highest *CCNB1* mRNA expression levels (Figure 3C) [32].

### Co-expressed Genes

Utilization of gene expression microarrays has made it clear that for a given gene investigated in a large enough data set other genes exist that display high similarity in expression pattern across samples. This co-expression of multiple genes is anticipated, given that functional processes and molecular pathways in an organism involve a highly organized interplay between multiple genes. Moreover, the co-expression of genes suggests that genes in a gene signature are often interchangeable as illustrated in a reanalysis of the van't Veer et al. [12] data set [39], indicating that it is the functional processes captured by a gene signature and not the individual genes that is of importance [22]. Recently, the approach of constructing metagenes or gene expression modules aimed at mimicking key biological processes in breast cancer has been utilized to identify gene sets with prognostic or treatment predictive power in breast cancer subgroups [4], [18], [19], [40]. These metagenes or gene expression modules are often based on identification of co-expressed genes to a specific prototype gene believed to represent the biological process of interest.

**Figure 4:**
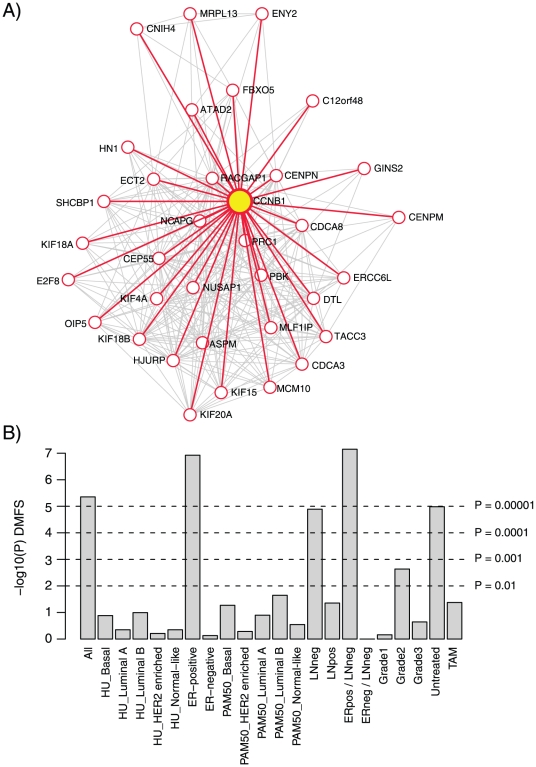
Identification of CCNB1 co-expressed genes and their association with outcome as a gene set. (A) GOBO analysis using the Co-expressed genes application identified 34 genes to be highly correlated with *CCNB1* in a 1751-sample subset of the full combined breast cancer data set (excluding Chin et al. cases). Iterative correlation analysis of the 35 genes showed that all genes were highly correlated to each other with at least 5 connections, as visualized by a Cytoscape V2.6.3 spring embedded network. Each connection is visualized as a line between two genes. *CCNB1* is highlighted in yellow. (B) PAM clustering into two groups of samples using the Sample Predictor (SP) application with the 35 genes in (a), followed by Kaplan-Meier analysis for 21 subgroups of the 1881-tumor set using DMFS as endpoint and 10-year censoring for 1379 cases with DMFS follow-up. Logrank P-values are shown as −log10(P-value).

The CG application of GOBO allows single genes to be used as prototype genes for identification of co-expressed genes by Pearson or Spearman correlation analysis in both subsets of the breast tumor data set (similar as GSA-Tumor) and the breast cancer cell line data set. The list of co-expressed genes can be further submitted to iterative correlation analysis, identifying which of the co-expressed genes that show tight co-expression with each other in an iterative process across the selected data set. The result of such an analysis could be a smaller cluster of genes that all are highly correlated with each other, representing a more distinct metagene or gene module (Fredlund et al., Manuscript in preparation). Using the CG application, we searched for genes highly co-expressed with *CCNB1* in a 1751-sample subset of the 1881-sample breast cancer data set (excluding Chin et al. [41] cases) (File S1). Using a Pearson correlation cut-off >0.6 combined with a gene expression log2 standard deviation cut-off >0.9, we identified 34 genes to be positively co-expressed with *CCNB1* (Table S1). When subjected to iterative correlation analysis one by one, all 35 genes showed at least 5 connections (Pearson correlation >0.6) with other highly co-expressed genes (Figure 4A, visualized using Cytoscape [42]). Functional analysis of the 35 genes using DAVID [43], [44] confirmed the strong association with cell cycle processes, specifically M-phase and mitosis (Table S2). *CCNB1* mRNA expression has been reported to be significantly and positively correlated with mRNA expression levels of *CENPE*, *AURKB*, *PLK1*, and *PLK4* in both breast tumors and breast cancer cell lines [32]. None of these genes were present in the list of 34 genes from the CG analysis, explained by lower correlations (*CENPE*: 0.47, *AURKB*: 0.42, *PLK1*: 0.39, *PLK4* 0.38) to *CCNB1* in our analysis. However, when performing a CG analysis in the Neve et al. cell line panel we found *PLK1*, *AURKB* and *CENPE* to be correlated (Pearson correlation >0.5) with *CCNB1* given a gene expression log2 standard deviation cut-off >0.4. Association with outcome for a derived co-expressed gene set may be further investigated by subsequent GSA-Tumor or SP analysis (Figure 4B, File S1), while association with biological processes could be made through usage of public functional annotation tools.

### Sample Prediction

A multitude of microarray-based gene signatures have been derived stratifying breast cancer into subtypes [1], [2], [24], identifying patients at risk [5], [6], [8], [9], [11], [12], and predicting response to therapeutic agents [3], [4]. However, it has become evident that gene signatures often represent the same biological processes [22], are associated with outcome only in subgroups of breast cancer [18], [19], and that validation of gene signatures needs to be performed in large independent data sets [45]. The SP application of GOBO is aimed at providing an interface for prognostic validation of simple gene signatures in similar subsets of the 1881-sample breast cancer data set as for GSA-Tumor. Currently, SP supports three forms of predictor designs; 1) PAM-clustering [46], 2) stratification by expression quantiles, and 3) correlative centroid prediction. SP analysis includes, similar to GSA-Tumor, Kaplan-Meier analysis, univariate and multivariate analysis of predicted groups, as well as correlation of predictor genes to functional modules (Figure 1B).

**Figure 5:**
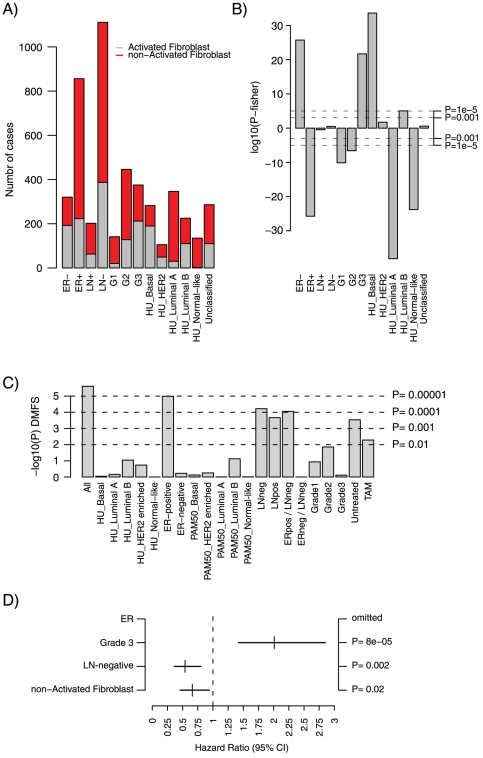
Sample Prediction analysis of the CSR gene signature in the 1881-sample breast cancer data set. (A) Composition of CSR classification groups for different clinical variables and Hu et al. gene expression subtypes in the 1881-sample set. (B) Association of the CSR activated fibroblast classification group with clinical variables and gene expression subtypes (Hu et al.). Y-axis display log10(P-value) from Fisher tests for each category. E.g., for the basal-like subtype a 2×2 table is generated containing number of basal-like tumors in the CSR activated fibroblast class, number of non-basal-like tumors in the CSR activated fibroblast class, number of basal-like tumors in the CSR non-activated class and number of non-basal-like tumors in the CSR non-activated class. Fisher P-values from tests with odds ratios <1 (negative association) are depicted as log10(P) (negative values on y-axis), whereas odds ratios >1 (positive association) are depicted as –log10(P) (positive values on y-axis). Results can be interpreted such that the CSR activated fibroblast class is associated with ER-negative tumors, tumors with histological grade 3, and tumors classified as basal-like or luminal B. (C) Association with outcome for CSR classification in subgroups of breast cancer using DMFS as endpoint and 10-year censoring. Samples in the 1881-sample set were stratified into two groups based on correlation to the CSR activated fibroblast gene signature, followed by Kaplan-Meier survival analysis in 21 subgroups using 1379 cases with DMFS follow-up. Logrank P-values are shown as −log10(P-value). (D) Corresponding multivariate analysis for ER-positive tumors (n = 554) using lymph node status and stratified histological grade (histological grade 1 and 2 vs. 3) as covariates, DMFS as endpoint and 10-year censoring. ER status is omitted from the multivariate analysis since all investigated cases are ER-positive.

Cancer invasion and metastasis represent the often-fatal steps in tumor progression, but remain poorly understood. Clearly, modifications in the tumor microenvironment involving not only tumor cells but also surrounding tissue, e.g., tumor-associated fibroblasts, are required for a tumor to become invasive and able to metastasize. Observations of histological similarities between the tumor microenvironment and normal wound healing have led to the hypothesis that tumors represent “wounds that do not heal” [47]. Fibroblasts represent a key component in wound healing, and in an attempt to identify a canonical gene expression signature of the fibroblast serum response to wound healing Chang et al. [48] reported a Core Serum Response (CSR) signature that was subsequently found to be associated with outcome in breast cancer patients [8]. In order to investigate the prognostic association of the CSR signature in subgroups of breast cancer we submitted the CSR activated fibroblast centroid [8] to SP analysis (File S1). Analysis of the association for the CSR signature with different clinical and molecular variables showed that the activated fibroblast class of tumors was predominantly associated with typical high-proliferative poor outcome classes in breast cancer, such as ER-negative tumors, histological grade 3 tumors, basal-like and luminal B classified tumors (Figure 5A and B). In contrast, the non-activated fibroblast class of tumors was associated with ER-positive tumors, histological grade 1 and 2 tumors, luminal A and normal-like classified tumors (Figure 5A and B). As expected from these results the CSR signature was associated with outcome for all tumors (Figure 5C).

**Figure 6:**
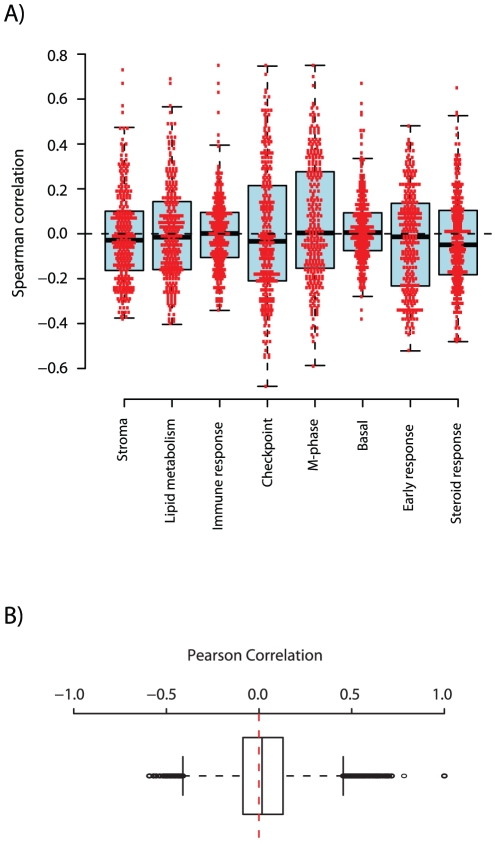
Correlation of genes in the CSR signature to different gene modules and pair wise co-expression. (A) For each gene module and gene in the CSR centroid (n = 304 matching), a Spearman correlation value is computed by comparing the expression pattern across all samples for a specific gene to the corresponding rank sum for each sample in the specific module. Red dots indicate actual correlation values. (B) Box plot of pair wise correlations (n = 46056 pairs) of 304 genes from the CSR centroid matching in the 1751-sample set used by the CG application, showing that the absolute majority of genes in the CSR gene signature is not co-expressed across a large set of breast tumors.

SP analysis also reveals if a signature is associated with outcome within subgroups of breast cancer stratified using clinical and molecular variables. Association with outcome for the CSR signature was mainly observed for ER-positive, LN-positive, LN-negative, untreated tumors, tamoxifen (TAM) alone treated tumors, or ER-positive/LN-negative tumors (Figure 5C). In further support, associations with outcome for CSR in ER-positive (Figure 5D), LN-positive and TAM tumors were supported by multivariate analysis using ER-status, LN-status and stratified histological grade as covariates when applicable. Taken together, the CSR SP and *CCNB1* GSA-Tumor analyses are consistent with results from meta-analyses of different prognostic gene signatures, including CSR, identifying proliferation as the major constituent of several signatures [19], [22]. These meta-analyses have shown the signatures to be predominantly associated with outcome in ER-positive breast cancers, whereas the signatures identify the majority of ER-negative breast cancers as poor outcome and cannot be used to further stratify ER-negative tumors into groups with different outcome. These findings reinforce that prognostic gene signatures in breast cancer need to be evaluated in a subtype-specific manner in sufficiently large sample sets [18], [19], [22], [49]. Interestingly, a majority of genes in the CSR centroid did not show clear co-expression with any of eight different co-expressed gene expression modules, including two proliferation modules (Checkpoint and M-phase) (Fredlund et al. Manuscript in preparation) (Figure 6A). Moreover, in exploratory analysis we found that genes in the CSR centroid did not show extensive co-expression when tested pair-wise in the 1751-sample subset used by the CG application (Figure 6B). Similarly, when dissecting several reported gene signatures Wirapati et al. found that when using only a subset of signature genes associated with proliferation, performance was identical or even improved for certain signatures [22]. This observation suggests that many prognostic gene signatures contain only a smaller fraction of genes actually associated with outcome, and a significant proportion of passenger genes that may perturb or even have a detrimental effect on the prognostic performance.

### Conclusions

GOBO is a convenient and user-friendly online tool for preliminary analysis of association with outcome for gene expression levels of single genes, sets of genes or gene signatures in a large public breast cancer microarray data set. Moreover, GOBO offers the possibility of investigation of gene expression levels in breast cancer subgroups and breast cancer cell lines for gene sets, as well as creation of potential metagenes based on iterative correlation analysis to a prototype gene. We have here demonstrated the usefulness of GOBO using *CCNB1* and the CSR gene signature as two examples for rapid online extraction of gene expression patterns, co-expressed genes, and survival analysis in a large breast cancer data set.

## Design and Implementation

GOBO is designed in three modules; a data set module, a web interface module, and a data processing module (Figure 1). The data set module contains normalized gene expression data sets, and corresponding annotation data compiled into R-data objects. The GOBO web-interface module is written using Perl CGI. In the data processing module, application specific Perl scripts are used to parse parameters passed from web-interface, and to launch application specific R-scripts for analysis. R-scripts perform all analyses using standard R packages, such as survival for outcome analysis. Upon completion, R-scripts return analysis results as files available from the web-server for download. In order to simplify extensive analysis comparisons, storage of analysis results and to provide plots as scalable vector graphics supporting incorporation in publications output from GOBO analyses are reported as text and pdf files.

## Availability and Future Directions

GOBO is freely accessible from the GOBO website http://co.bmc.lu.se/gobo. The design and implementation of GOBO facilitate easy incorporation of additional query functions and applications, as well as additional data sets irrespective of tumor type and array platform in the form of precompiled R-data sets. Future integration of data sets with additional information such as mutation status, copy number alterations or methylation data may further enhance the usefulness of GOBO.

## Supporting Information

Table S1 **Result of Co-expressed genes analysis for** ***CCNB1*** **.** An Excel table listing genes co-expressed with *CCNB1* in the combined breast cancer data set.
(XLS)

Table S2 **Result of functional analysis of genes co-expressed with CCNB1 using DAVID.** An Excel table showing the results from functional analysis using DAVID for *CCNB1* and 34 co-expressed genes.
(XLS)

File S1 **Supplementary Methods.** A Word document describing the preprocessing of the breast tumor and breast cancer cell line microarray data sets, and settings for described GOBO analyses.
(DOCX)

File S2 **Investigation of data set bias in the combined 1881-sample data set.** A PDF file with figures S1 to S5 displaying result of PCA analysis of the 1881-sample set, examples of gene expression levels for Affymetrix probe sets matching ER and HER2 across clinical and molecular annotations, and clustering of the 1881 cases using the PAM50 gene set.
(PDF)
