# Supplementary material for: GOBO: Gene Expression-Based Outcome for Breast Cancer Online
Source: PLoS One. 2011 Mar 21;6(3):e17911. doi: 10.1371/journal.pone.0017911 (PMC3061871; doi:10.1371/journal.pone.0017911)
Supplement: File S1 — Supplementary Methods. A Word document describing the preprocessing of the breast tumor and breast cancer cell line microarray data sets, and settings for described GOBO analyses. (DOCX) [file pone.0017911.s003.docx]

**Preparation of Neve et al. cell line data**

Normalized gene expression data were obtained from Neve et al. [23]. Based on the na.30 probe set descriptions for the Affymetrix U133A, array control and cross-hybridizing probes, identified as probe sets referencing multiple Entrez Gene id's or by a x_at probe name suffix, were removed. Log2 expression values for remaining probes were merged based on Entrez Gene id resulting in a data set representing 12,242 unique gene identifiers. In parallel, to the gene-merged data set an Affymetrix probe centered data set was created by mean-centering log2 expression values for the 18223 Affymetrix probe sets passing filtering.

**Preparation of Affymetrix 1881-sample breast cancer data set**

CEL files for all data sets except GSE2034 and GSE5327 were obtained from public repositories. Data for GSE2034 and GSE5327 were obtained from GEO [13] as MAS5 normalized intensity data scaled to 600 as target. CEL files were normalized using MAS 5.0, with 600 as target. Based on the na.30 probe set descriptions for the Affymetrix U133A array control and cross-hybridizing probes, identified as probe sets referencing multiple Entrez Gene id's or by a x_at probe name suffix, were removed. Log2 expression values for remaining probes (n=18223) were merged based on Entrez Gene id resulting in a gene-merged data set representing 12,242 unique gene identifiers. Next, genes were mean-centered across the full data set. In parallel, to the gene-merged data set an Affymetrix probe centered data set was created by mean-centering log2 expression values of the 18223 filtered Affymetrix probes. Clinical annotations were obtained from GEO [13], publications (GSE2034) or Chin et al. [41]. For data set GSE2603, metastasis-free survival was interpreted as DMFS. For the Chin et al. data set, the distrec variable was interpreted as DMFS.

**Classification of breast cancer data set**

Samples in the combined Affymetrix data set were classified according to gene expression subtypes reported by Hu et al. [29] and Parker et al. (PAM50) [24]. Gene expression centroids from Hu et al. and Parker et al. were matched based on gene identifier to the gene-merged Affymetrix data set, and correlation to centroids were calculated using Pearson correlation. A correlation cut-off of 0.2 was used to call samples. Samples with highest correlation <0.2 to any centroid were denoted unclassified.

**Settings for CCNB1 analysis in GSA-Tumor, Co-expressed genes and Sample Prediction**

**GSA-Tumor settings**

The input gene set consisted of *CCNB1*. The full data set (All tumors) was stratified into three equally sized quartiles based on *CCNB1* mRNA expression levels. Distant metastasis free survival (DMFS) was selected as endpoint, with 10-year censoring. ER status, lymph node status and stratified histological grade (grade 1 and 2 versus 3) were used as covariates in multivariate analysis. Gene symbols were used as input type.

**Co-expressed genes settings**

The input gene set consisted of *CCNB1*. The full data set (All tumors) excluding the Chin et al. data set was used to identify co-expressed genes. Genes passing a standard deviation cut-off of log2(ratio) >0.9 were subjected to correlation analysis. Using Pearson correlation, genes with correlation >0.6 to *CCNB1* (n=34) were identified as co-expressed. *CCNB1* and the 34 co-expressed genes were subjected to iterative correlation analysis identifying which of the 35 genes that showed correlation >0.6 to at least 5 other genes in the set of 35 genes.

**Sample Prediction PAM analysis of genes co-expressed with *CCNB1***

The input gene set consisted of *CCNB1* and 34 other genes identified as co-expressed with *CCNB1* by the CG application. The full data set (All tumors) was stratified into two groups using PAM clustering. DMFS was selected as endpoint, with 10-year censoring.

**Settings for CSR analysis using Sample Prediction**

Input consisted of the CSR activated fibroblast centroid [8] updated for probe annotations. The full data set (All tumors) was stratified into two groups based on Spearman correlation to the CSR centroid using gene symbols as input types for matching. A correlation cut-off >0.1 was used to call samples as having an activated fibroblast signature or alternatively a non-activated signature. DMFS was used as endpoint, with 10-year censoring.
