# Supplementary material for: GOBO: Gene Expression-Based Outcome for Breast Cancer Online
Source: PLoS One. 2011 Mar 21;6(3):e17911. doi: 10.1371/journal.pone.0017911 (PMC3061871; doi:10.1371/journal.pone.0017911)
Supplement: File S2 — Investigation of data set bias in the combined 1881-sample data set. A PDF file with figures S1 to S5 displaying result of PCA analysis of the 1881-sample set, examples of gene expression levels for Affymetrix probe sets matching ER and HER2 across clinical and molecular annotations, and clustering of the 1881 cases using the PAM50 gene set. (PDF) [file pone.0017911.s004.pdf]

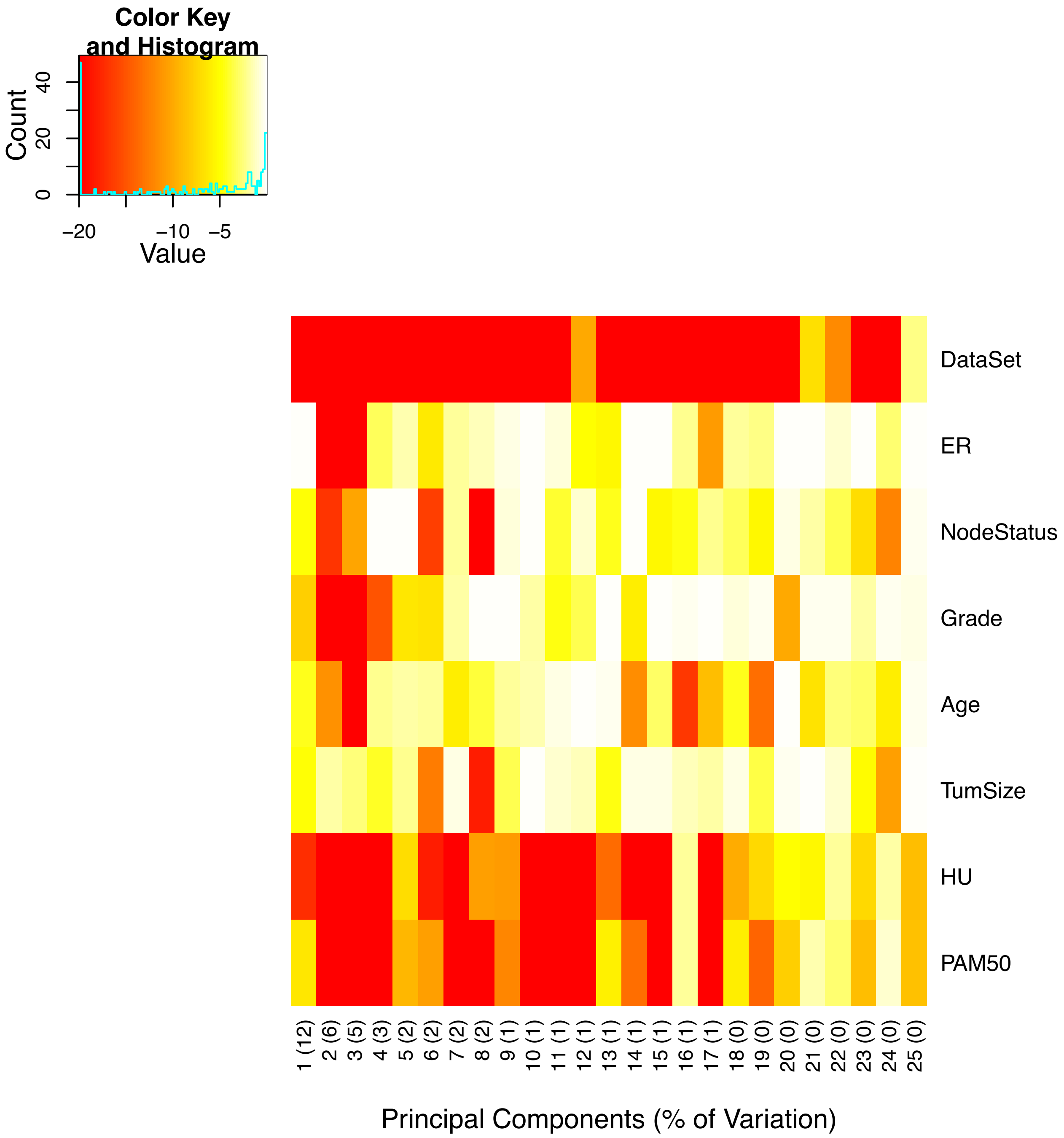

Figure S1. PCA analysis to investigate associations between variation in the 1881-sample gene expression data set merged on Entrez Gene ID and various sample annotations. The principal components are shown horisontally, with the fraction of the variation associated with each principal component shown within parenthesis. The evaluated sample annotations are shown vertically. Each principal component was associated to each sample annotation using a linear model fit. The linear model fits were evaluated using the F-statistic and log10 of the P-values are pseudocolored in the plot. Only the first 25 principal components are shown.

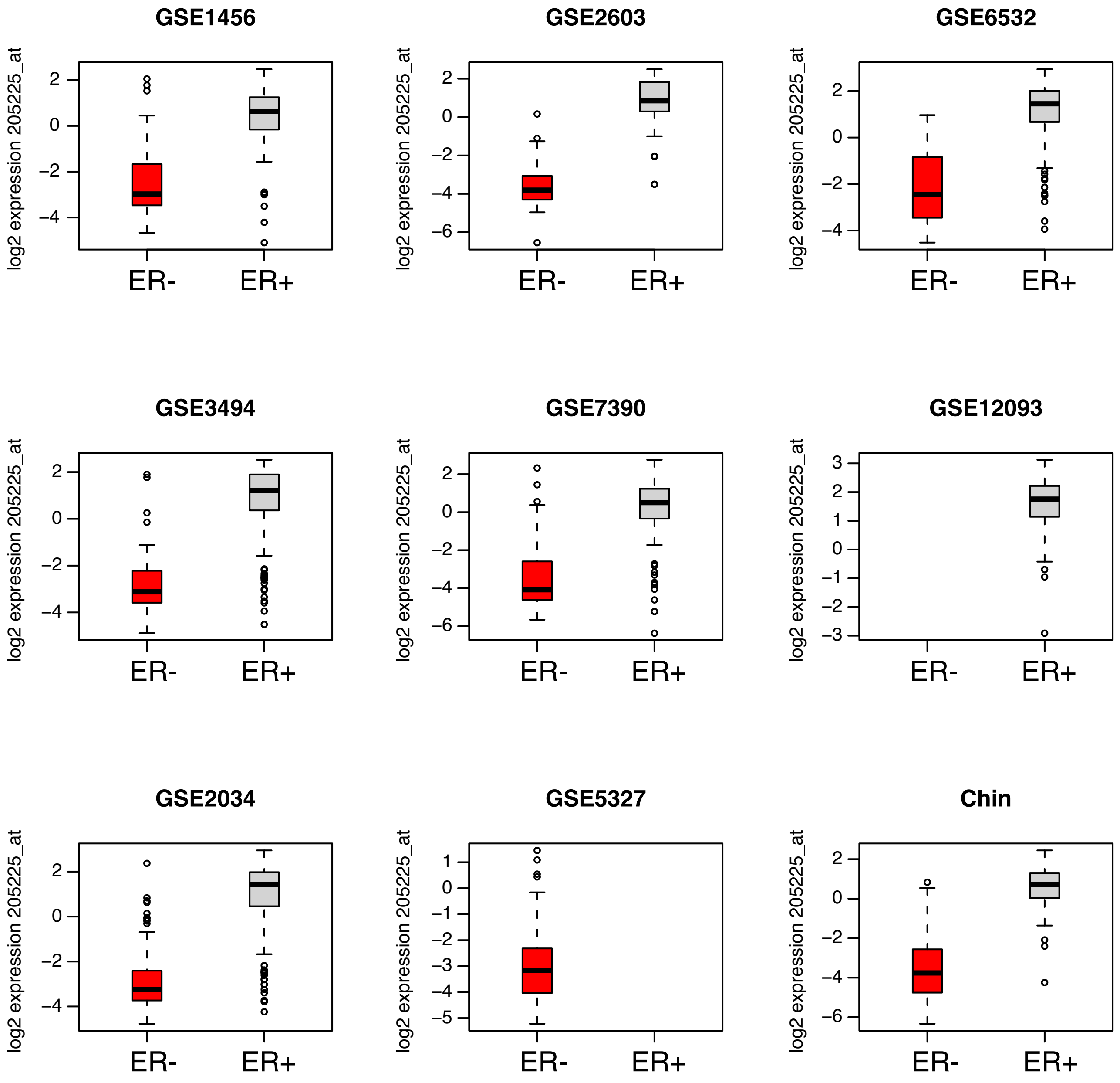

Figure S2. Log2 gene expression values for Affymetrix probe 205225_at mapping to *ESR1* across nine different data sets with ER-status included in the pooled 1881-data set. Affymetrix probe 205225_at have been reported to have the highest concordance with biochemical ER-status. (Karn et al. [21]).

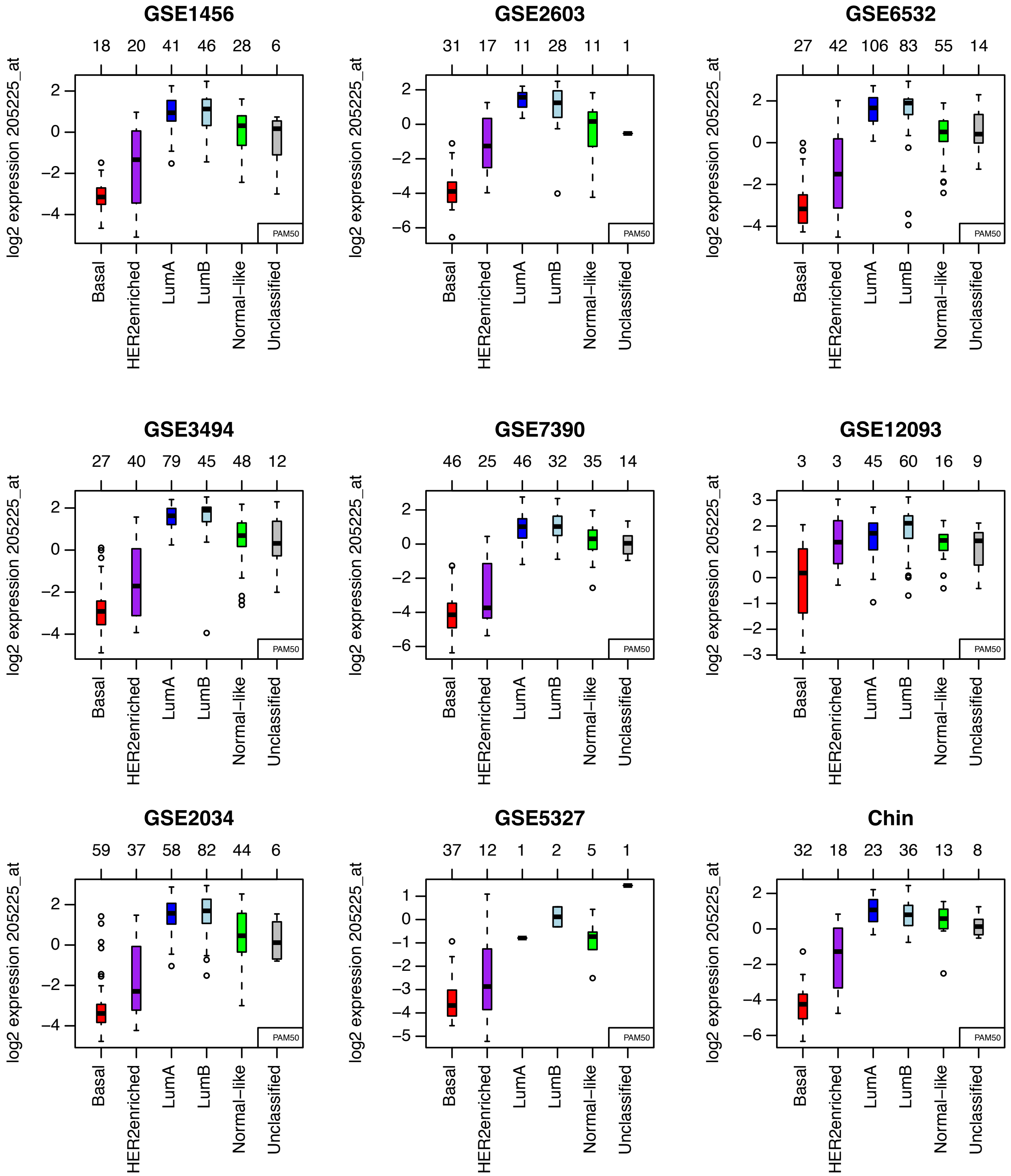

Figure S3. Log2 gene expression values for Affymetrix probe 205225_at mapping to *ESR1* across nine different data sets included in the pooled 1881-data set. Tumors are grouped according to PAM50 molecular subtype classification [26]. Affymetrix probe 205225_at have been reported to have the highest concordance with biochemical ER-status. (Karn et al. [21]).

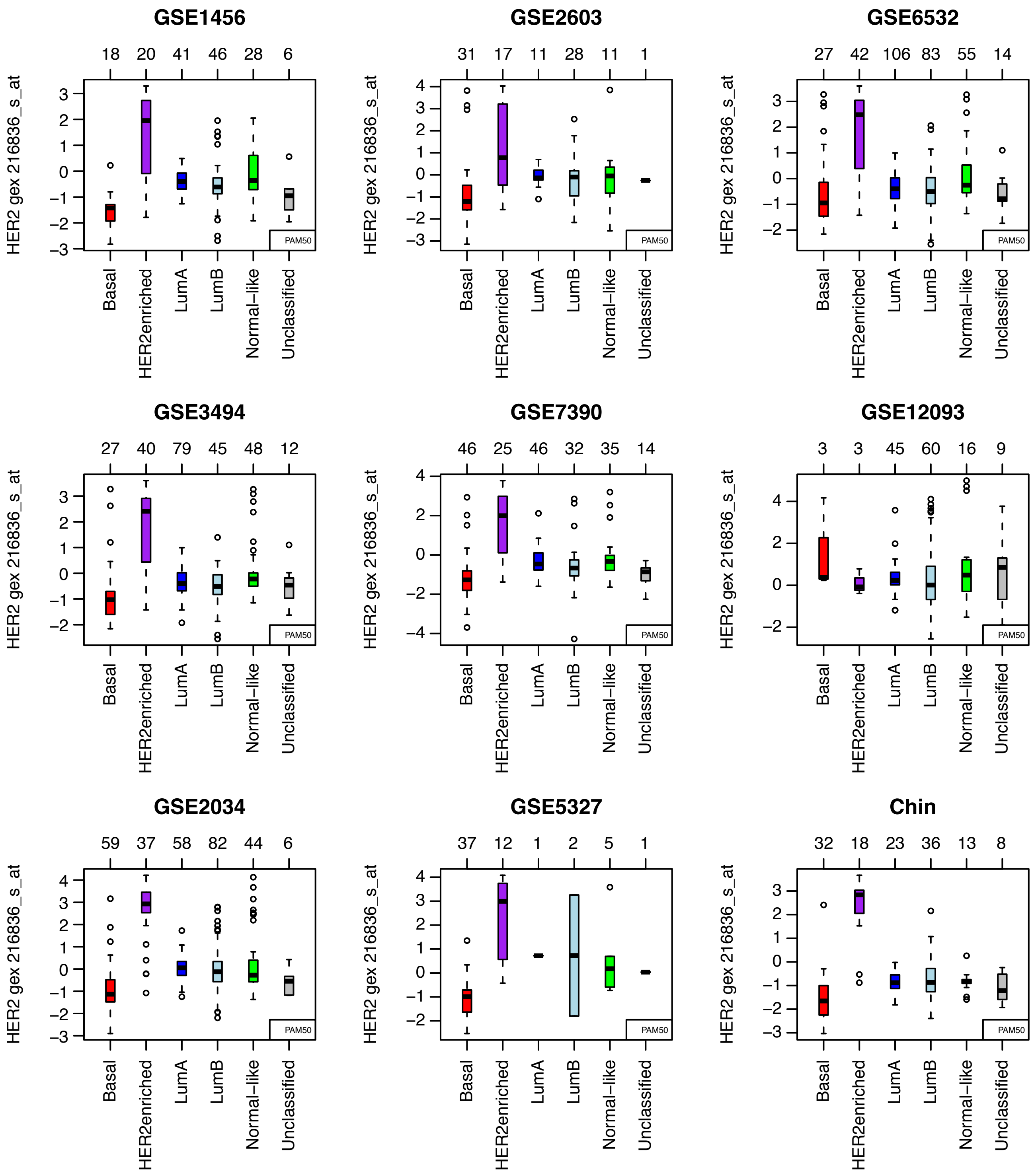

Figure S4. Log2 gene expression values for Affymetrix probe 216836_s_at mapping to *HER2* across nine different data sets included in the pooled 1881-data set. Tumors are grouped according to PAM50 molecular subtype classification [26]. Affymetrix probe 216836_s_at was used by Karn et al. [21] to derive data driven cut-offs from gene expression data for clinical subtype classification.

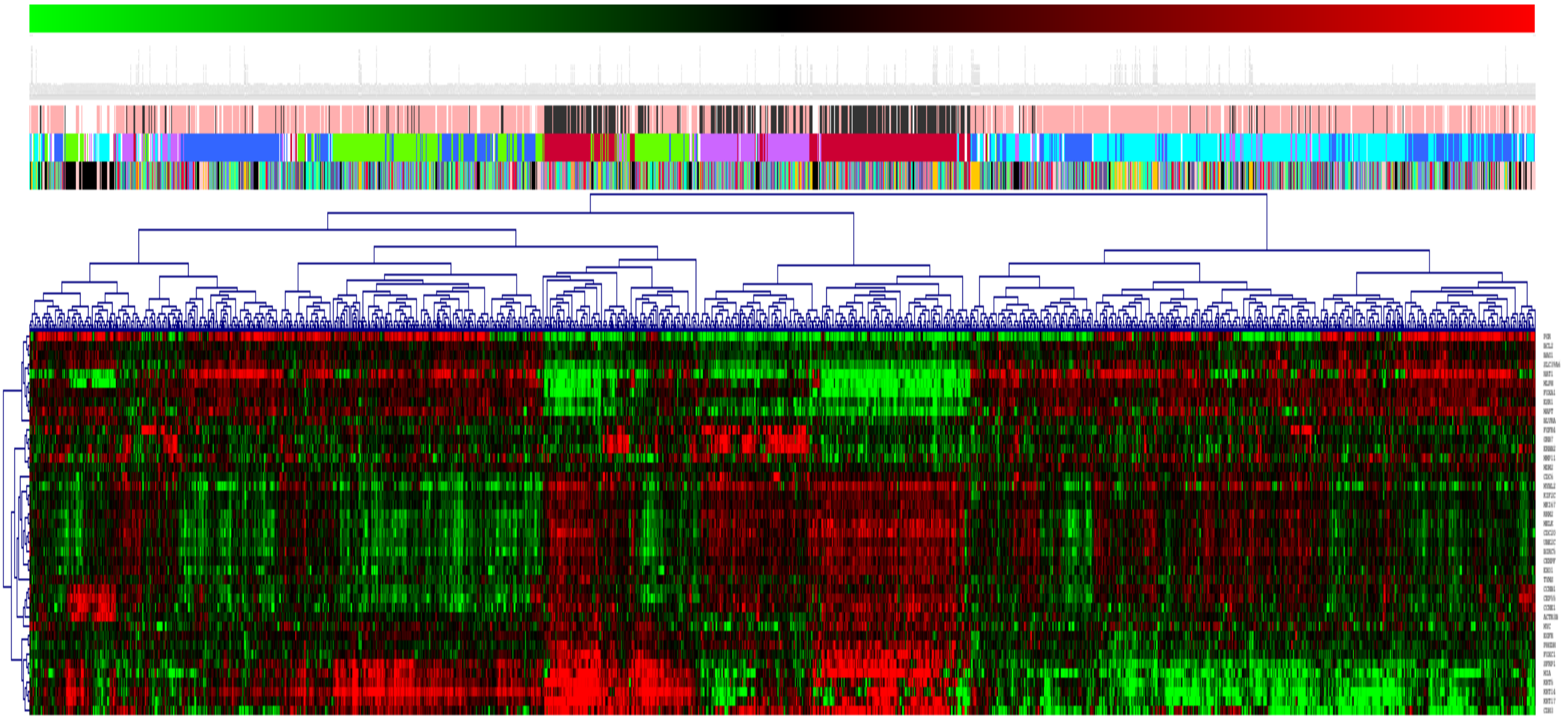

Figure S5. Clustering of PAM50 genes in the 1881-sample data set. The lower annotation bar indicates data set affiliation for each sample (11 different data sets present). The center annotation bar shows PAM50 centroid classification, basal-like (red), HER2 enriched (purple), luminal A (blue), luminal B (light blue) and normal-like (green). Top annotation bar shows ER-status for samples with available annotations; ER-negative (black) and ER-positive (pink). Clustering performed using Pearson correlation and complete linkage.
